# Thermal-mediated modulation of binary supramolecular self-assembly from phase separation to co-crystallization at the liquid–solid surface

**DOI:** 10.1039/d5sc06698k

**Published:** 2026-01-13

**Authors:** Fang Chen, Jun He, Attia Shaheen, Yi Hu, Shern-Long Lee

**Affiliations:** a Institute for Advanced Study, Shenzhen University Shenzhen Guangdong 518060 China sllee@szu.edu.cn; b School of Chemistry and Chemical Engineering, Anqing Normal University Anqing Anhui 261433 China hu.yi@aqnu.edu.cn

## Abstract

Significant research in materials chemistry has focused on the design and fabrication of organic materials and their self-assembled architectures for a wide range of applications, such as organic transistors, photovoltaic cells, and surface functionalization, to name just a few. For binary supramolecular systems, however, the increased complexity that involves hetero-molecular interactions often leads to challenges, for instance, undesired phase segregation. Using scanning tunnelling microscopy (STM), we show that thermal activation (from 25 °C to 60 °C) can drive a transition from phase separation to thermodynamically stable co-crystallization for a host–guest system comprising trimesic acid and a tetrathiafulvalene derivative. Our STM data revealed that the co-crystals varied from the chicken-wire type to a flower type as a function of annealing temperature (from 60 °C up to 80 °C). Their molecular interactions and adsorption energy and thus the corresponding stability constitute the energy landscape, which is derived from force-field simulations. This transformation could be governed by the modulation of molecule–substrate interactions, intermolecular bonding, and hetero-molecular attractions, offering a thermally tuneable route toward supramolecular co-assemblies.

## Abstract 2

This study elucidates the thermal-mediated modulation to form vertically stacked bilayers in a binary supramolecular system, which otherwise would form phase-separated domains on a graphite surface.

## Graphical abstract

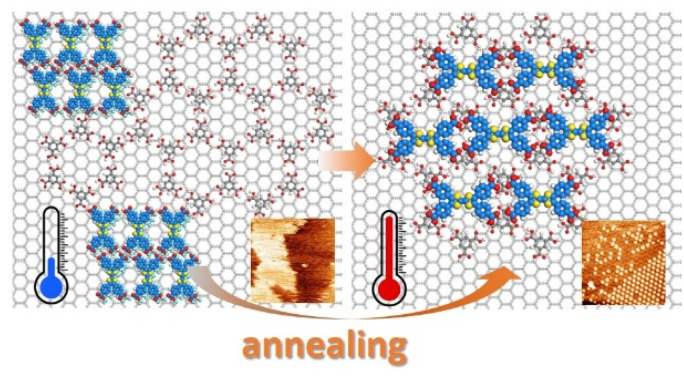

## Introduction

The construction of functional materials *via* the bottom–up approach demands rationally designed molecules and meticulous control over these nanoarchitectures.^1,2^ To accomplish this objective, self-assembly is increasingly utilized as a powerful strategy by which molecules can undergo error correction and self-healing, reaching a well-ordered state at thermodynamic equilibrium. Such surface-confined motifs with a coherent molecular orientation are closely related to crystal engineering and can further be integrated into molecular-based devices, including sensing, organic field effect transistors (OFETs), organic light-emitting diodes (OLEDs), organic solar cells (OSCs), and perovskite solar cells (PSCs), to name just a few.^3,4^

The paradigm of research interest in surface molecular assemblies has recently shifted from self-assembly monolayers (SAMs) to a few layers including bilayers.^5^ Simultaneous control over the in-plane and out-of-plane interactions of molecules is vital to generate such hierarchical architectures. On the other hand, methodological developments allow thin-film formation in a layered fashion, including Langmuir–Blodgett and layer-by-layer deposition techniques. The lateral stability of molecular assemblies at surfaces is generally maintained by van der Waals, interactions, π–π stacking, electrostatic attractions, metal–ligand coordination, hydrogen bonding, halogen bonding and ionic interactions;^6^ most of the vertical packing is driven by π–π interactions of molecular aromatics.^7^ For binary systems, the level of complexity increases. Although the same supramolecular interactions are involved, the final output can suffer from some disadvantages, for example, segregated stacking. When interactions of individual molecules are stronger than those between the two types of molecules, it results in phase separation to achieve the maximal stable energy for a system (*vice versa*).^8–10^

The exploration of cutting-edge techniques or methods for generating co-crystals merits special attention as such hybrid structures may impart unique functions (*e.g.*, Moiré patterns and their properties). Also, it provides the exciting possibility for studying charge transport in a heteromolecular junction, especially for the host–guest complexation of donor–acceptor hybrids^11^ For these systems, crucial factors include size, shape, symmetry, and geometry matching between the host and guest. In general, the host molecules constitute a template with rich voids that allow guest incorporation. The well-known building blocks of host templates include trimesic acid (TMA), 1,3,5-tris(4-carboxyphenyl)benzene (BTB), *p*-terphenyl-3,5,3′,5′-tetracarboxylic acid (TPTC), or hexadecyloxy substituted dehydrobenzo[12]-annulene (DBA-OC16).^12^ The guests are generally coronene or fullerene having relatively higher mobility than the template molecules on a surface. The host can immobilize these guest species. The guests can also be other materials like metal clusters or nanoparticles.^13–15^ Subtle interactions balance the intricate surfaces establishing the so-called template-assisted method. To date, up to four-component patterns have been achieved on a surface.^16^ Despite much exploration in the past, it remains challenging to control the output between phase separation and co-crystallization for a system. Externally applied stimuli including thermal or electric field have been used to approach this goal.^17,18^ Previously, we reported synergistic stimuli, combining temperature and electric field for cooperative tailoring of supramolecular phase transformations using TMA as a model system and by means of scanning tunneling microscopy (STM) equipped with a temperature controller.^19^

Due to their remarkable electronic properties, tetrathiafulvalene (TTF)^20^ and its derivatives are promising candidates for molecular devices. Herein, a TTF derivative is chosen as the test-ground molecule. On the other hand, TMA representing the simplest structure with *C*_3v_ symmetry, has been widely used to construct hydrogen-bonded organic frameworks (HOFs). In this work, using STM we investigated the TMA–TTF mixing behaviour at the octanoic acid (OA)–highly oriented pyrolytic graphite (HOPG) interface. TMA and TTF represent molecules modified with carboxylic groups. They can lead to hydrogen bonding in their assemblies where the selectivity and directionality of hydrogen bonds are essential for steering self-assembly. We elucidate how concentration, thermal and solvent effects, and sample-preparation procedures influence the resulting outcome that can be either phase separation or co-crystal surfaces. Our experiments revealed that thermal stimuli are crucial for the formation of host–guest co-assemblies where TTFs constitute the upper layer atop TMA templates. Force field simulations were utilized to unveil the adsorption energies and corresponding stability of the polymorphic packings of TMA, TTF, and the TMA–TTF co-crystals. Finally, the plausible mechanism is presented, providing new insight into the binary component supramolecular systems at the solution–solid interface.

## Results and discussion

**Fig. 1:**
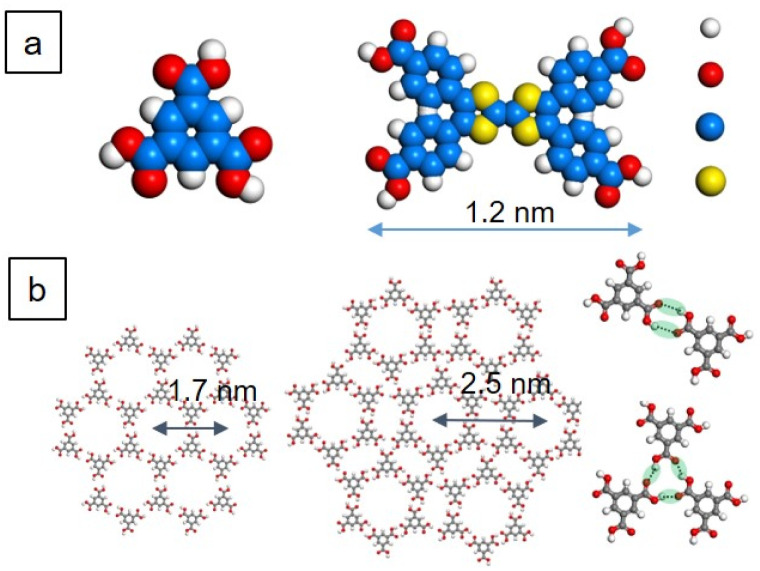
(a) Chemical structures of TMA and TTF and (b) models of TMA self-assemblies. In (a), the white, red, blue, and yellow dots refer to H, O, C, and S atoms, respectively.

Fig. 1a shows the chemical structures of TMA and TTF. Although organic molecules tend to form compact packings to realize minimum global energy, here TMA serves as templates as TMAs are well known to form extended hydrogen-bonded porous networks.^21^ The commonly found motifs are chicken-wire (CW) and flower structures stabilized by two different intermolecular hydrogen bonds between carboxyl groups (Fig. 1b). The center-to-center distances between neighboring voids are *ca.* 1.7 and 2.5 nm for the CW and flower motifs, respectively. Both exhibit periodic porous structures with 1.0 nm-diameter cavities. The size of TTF is *ca.* 1.2 nm in its long axis slightly larger than that of the TMA voids. Therefore, it is interesting to investigate their host–guest co-assembly.

**Fig. 2:**
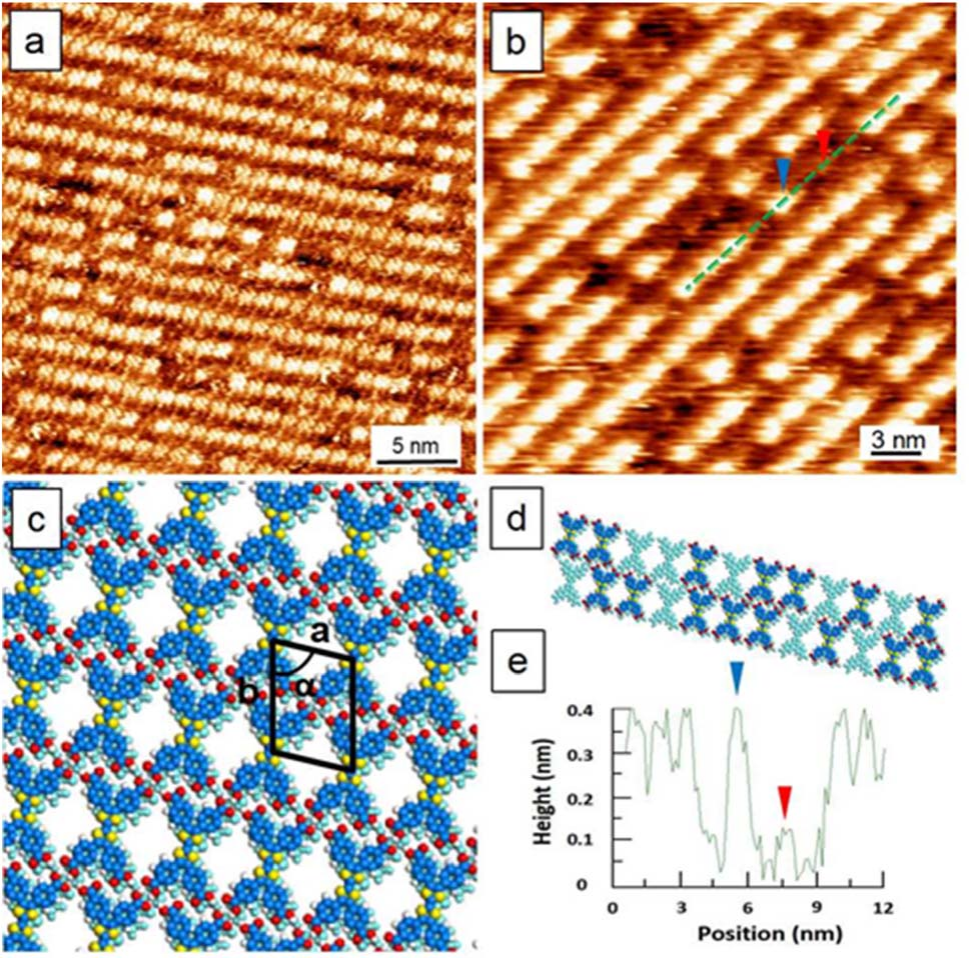
STM images, models, and sectional profile of TTF self-assemblies. (a and b) The self-assemblies of TTF at the OA/HOPG interface (0.1 M). Imaging conditions: for (a) *E*_bias_, −0.80 V; *i*_tunneling_, 500 pA; for (b) *E*_bias_, −0.88 V; *i*_tunneling_, 550 pA; (c and d) packing models of the structurally optimized packing and real bilayer with defects. The top and bottom layer are modelled in dark and light blue, respectively. Unit-cell parameters: *a =*1.5 nm, *b =*1.8 nm, and *α* = 78°; (e) a cross-sectional height profile runs along the green line in (b). Two arrows indicate the bilayers with *ca.* 0.3 nm height difference.

Fig. 2a and b displays the typical STM images of the TTF self-assembly at the OA/HOPG interface where the rich defects suggest that the packing is unlikely to be a monolayer. A cross-sectional height profile along the green line indicated in Fig. 2b is plotted (Fig. 2e), revealing a height difference of *ca.* 0.3 nm, consistent with layered TTF structures. Moreover, time-dependent STM monitoring (Fig. S1, SI) reveals the dynamic nature of this interface: the initial monolayer domains were observed to be metastable, gradually disappearing during the scanning process. This dynamic evolution confirms that the observed stacking is a genuine physical adsorption phenomenon, a behavior intrinsic to TTF analogues, which tend to aggregate into bilayers as reported in recent studies.^22–24^ The observed TTF packing features a linear packing topologically comparable to the oblique phase of BTB. The similar packing arising from different molecular tectons may result from the two-fold symmetric arrangement of the carboxylic groups of either a TTF or the dimer of BTBs (*R*_2_^2^(8)).^25,26^Fig. 2c and d shows a tentative model of structurally optimized packing and a real bilayer with defects. Note that the defects appear as vacancies, resulting from the absence of some single TTF molecules. Besides, the positional difference of these vacancies between the bottom and top TTF layers is strong evidence of bilayer formation. An unstable monolayer appeared at a high sample concentration (0.1 M), indicating its intrinsically kinetic nature. Our results are consistent with literature reports indicative of the strong tendency of TTFs to stack vertically into bilayers.^20,22,27,28^ Due to the existence of rich defects and imperfect packing, lattice-structure information was estimated by the tentative model.

**Fig. 3:**
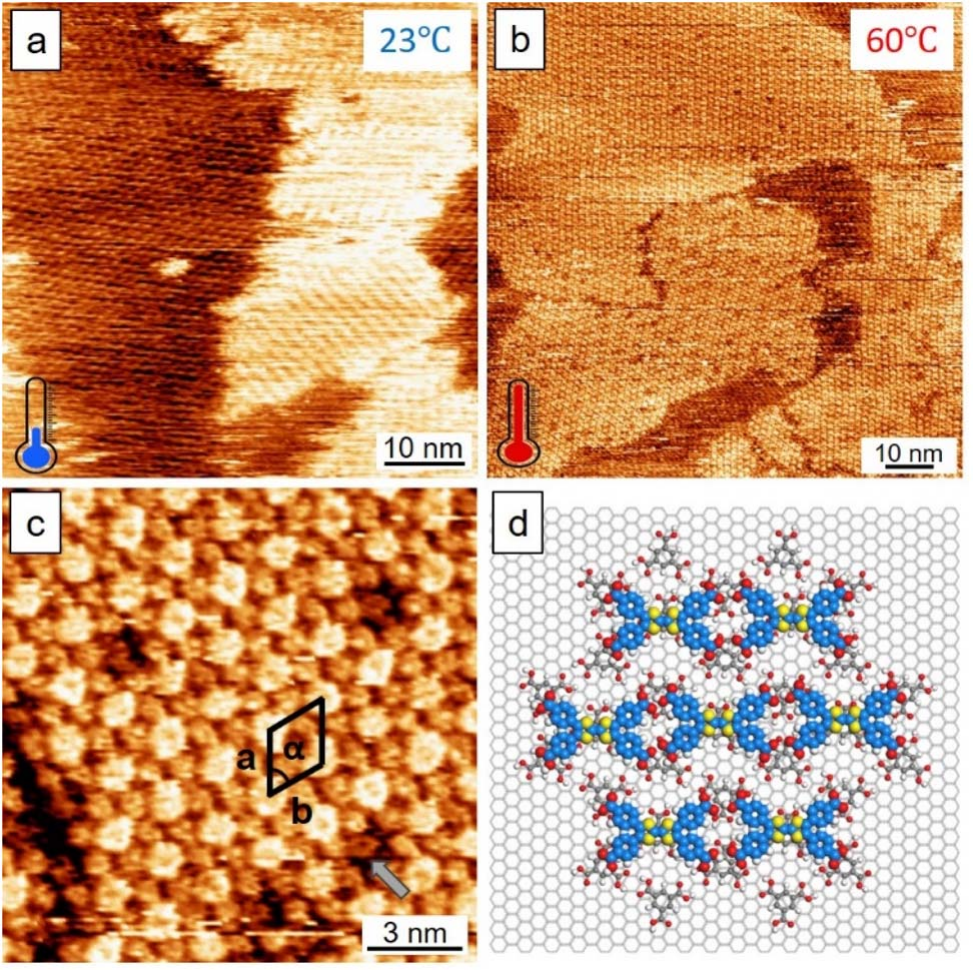
STM images of the TMA and TTF mixture on the HOPG surface. (a) Without thermal treatment: phase separation of TMA and TTF. (b and c) Large- and small-scale images of a surface with thermal annealing. (d) The co-crystal model. Unit-cell parameters of *a*, *b*, and *α*: 1.7 ± 0.2 nm, 1.7 ± 0.2 nm, 61 ± 3°. An arrow in (c) indicates the trapped TMA. Imaging conditions: for (a) *E*_bias_, −0.80 V; *i*_tunneling_, 300 pA; for (b) *E*_bias_, −0.80 V; *i*_tunneling_, 350 pA; for (c) *E*_bias_, −0.80 V; *i*_tunneling_, 550 pA.

Before mixing, the different adsorption and self-assembly abilities of TMA and TTF were tracked individually. We used the same sample-preparation procedure including sample concentration (0.1 M with OA as the solvent) and sonication (30 min prior to deposition onto HOPG). A histogram revealed that there were two clearly distinguishable time intervals where TMA self-assembled networks appeared faster than those of TTF by at least 20 min (Fig. S2, SI). The 1 : 1 mixture (volume ratio) of TMA : TTF (0.1 M) led to a surface entirely covered with TMA, implying the superior kinetics and thus adsorption ability of TMA than those of TTF at an earlier stage. Phase separated domains appeared after hours indicative of their competitive adsorption on the HOPG surface. Fig. 3a presents the phase separation where TMAs and TTFs exhibit honeycomb and striped domains (left and right in the image) that directly contact the HOPG surface, respectively.

Temperature is a key parameter influencing molecular self-assembly. Elevated temperature may assist in desorbing solvent or weakly adsorbed guest molecules that compete for surface adsorption, thereby promoting a more stable interface conducive to organized assembly. Further *ex situ* heating (60 °C, 30 min) over the surface yielded co-crystals that would otherwise be inaccessible without thermal stimulation (Fig. 3b). The images were taken at room temperature with a droplet of OA solvent covering the surface to maintain a liquid–solid-interface condition. The co-crystals feature Moiré superlattices. A high-resolution STM image reveals TTFs adsorbed atop the honeycomb TMA template (Fig. 3c). Fig. 3d shows the co-crystal model. The TTFs were stabilized by both horizontal TTF–TMA hydrogen bonding and vertical π-stacking with trapped TMAs (*e.g.*, excess of TMA).^29^ It is also possible to optimize surface coverage and minimize defects in the host–guest vertical stacking by systematically tuning temperature. Remarkably, the TMA template and thermal effect reduced the defects in the assembly, compared to the normal self-assembled bilayers of TTF (Fig. 2). The host–guest vertical stacking is reminiscent of the layer-by-layer deposition method that involves sequentially depositing the second layer on top of the first template.^30^ A parallel test carried out based on this approach, however, shows that without heat stimuli, efficiency of yielding such co-crystals would be limited. A surface obtained by the layer-by-layer deposition of TMA and TTF is shown in Fig. S3, SI. For comparison, the sample solutions were prepared without thermal treatment. From this surface, we observed that TTFs tended to pack into dimers and randomly adsorb on the honeycomb TMA template, significantly proving the thermal effect on the system. This exercise suggests that thermal input, rather than deposition sequences, is the key to obtaining the host–guest coherent stacking.

The sectional profile in Fig. S4, SI shows the height difference of the TTF–TMA co-crystal (*ca.* 0.1 nm). Such a height difference reflected not only topography but also electronic information of the conduction of the two different molecules.^7^ Thus, we reason TTF to be the upper layer based on the following reasons: (1) structural mismatch prevents the inclusion of the guest (Fig. 1): the longitudinal span of TTF (∼1.2 nm) exceeds the pore diameter of the TMA network (∼1.0 nm); (2) the pores were occupied by trapped TMAs during annealing of excess TMAs;^29^ (3) TTFs form their own domains and cannot readily go into the TMA pores either by simple mixing (Fig. 3a) or by layer-by-layer deposition (Fig. S3, SI) of TMAs and TTFs. Importantly, the apparent height value observed here (0.1 nm) correlates well with that of the 1,3,6,8-tetrakis(1-butyl-1*H*-1,2,3-triazol-4-yl)pyrene (TP)–TMA bilayer co-crystal reported in the literature,^29^ exceeding the height observed for a single-layer (*ca.* 0.05 nm).

**Fig. 4:**
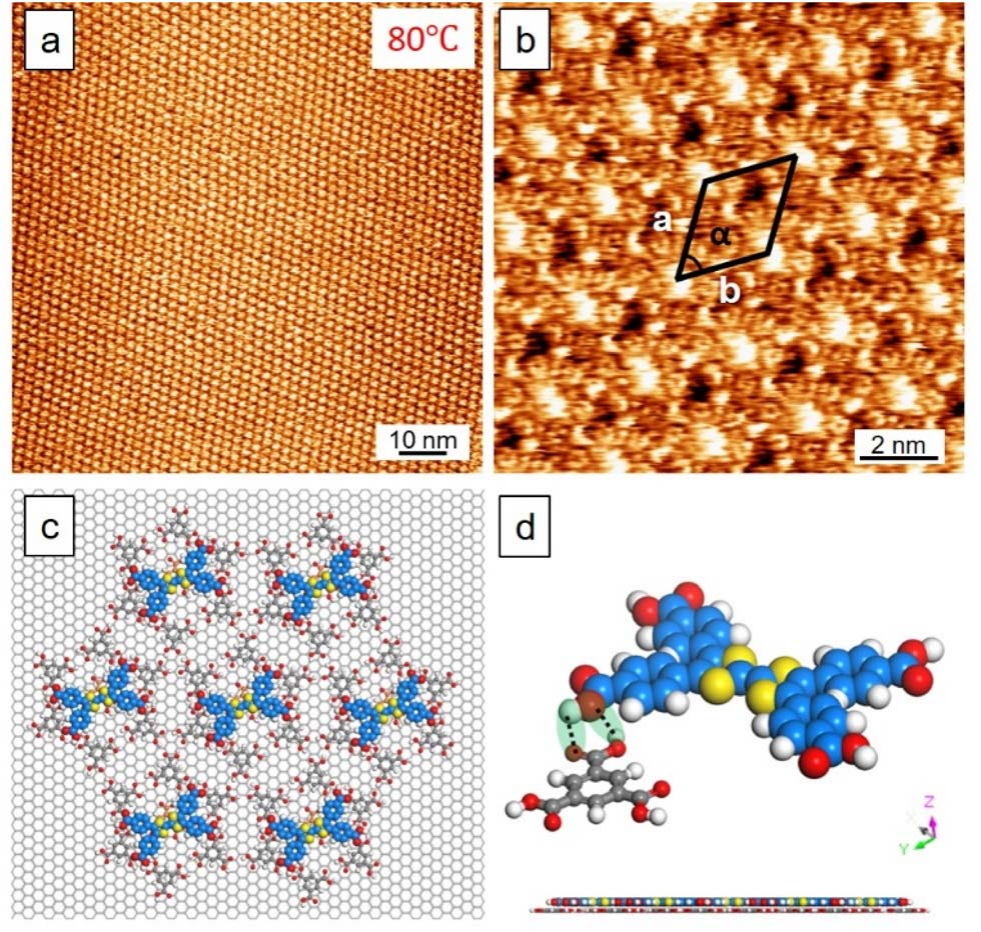
STM images of the TMA–TTF co-crystal at the OA/HOPG interface obtained after annealing at 80 °C. (a and b) Large, small-scale images. Unit-cell parameters of *a*, *b*, and *α*: 2.5 ± 0.1 nm, 2.5 ± 0.2 nm, 60 ± 2°. (c andd) The packing model of the co-crystal. Top and side views of the packing show the out-of-plane hetero–molecular hydrogen bonding. Imaging conditions: for (a) *E*_bias_, −0.80 V; *i*_tunneling_, 100 pA; for (b) *E*_bias_, −0.80 V; *i*_tunneling_, 400 pA.

To future elevate the annealing temperature (80 °C, 90 min), the surface turned into a new type of co-crystals (Fig. 4). A detailed analysis of such a pattern unveiled TTFs adsorbed on specific sites of the flower template of TMA. Presumably, the guests were stabilized by out-of-plane hetero–molecular hydrogen bonding as schematically depicted in Fig. 4c and d where both top-and side-view models are shown (height profile, see Fig. S4, SI). Besides, simultaneous stabilization by π attraction with the trapped TMAs is also possible. The well-ordered co-assembly is also ascribed to the increased temperature that may aid in aligning molecules along surface potentials or lattice constraints, enhancing epitaxial ordering and reducing domain boundaries. Having explored the thermal impact, the concentration effect was also examined. The low concentration of sample solutions did not influence our conclusion. Interestingly, the co-crystal can appear in a sub-monolayer fashion yet the TMA–TTF assemblies were still vertically stacked after thermal treatment, as shown in Fig. S5, SI. The impact of solvent together with the thermal effect on the system was also investigated. Using heptanoic acid (HA) as the solvent for dissolving both samples and after thermal treatment (30 min), the mixed surface yielded the flower type co-crystal pattern when the temperature was higher than 40 °C (Fig. S6, SI). For HA as the solvent, the CW type co-crystal was not a preferred form indicating a solvent effect. Temperature changes may alter solvent properties such as viscosity and dielectric constant, which in turn influence solvation dynamics and interaction strengths between assembling units. A systematic examination for host polymorphic forms also led to the same conclusion (Fig. S7 and S8 SI).

Thermal annealing is a common technique utilized to improve molecular packing, remove defects, and trigger chemical reactions during or after molecular depositions, for either SAMs or binary molecular systems.^31–35^ Generally, it enables better crystallinity and layered stacking as well as improved electronic transport properties. In our work, we also showed that such thermal effect would be helpful for steering the up-layer TTF self-assembly on a TMA template layer (the guest size is slight larger than the pore diameter of TMA). Moreover, interestingly, it can be efficiently used to control phase separation and co-crystal formation in binary molecular systems. In the literature, heat-induced phase-transition experiments are based on spontaneous supramolecular host–guest co-assembly and stimuli-sensitive hosts that can undergo phase transformations upon stimulation. For example, high temperature generates a close-packing host that squeezes guests embraced in the cavities of a porous host.^17^ For our present work scenario, the mechanism is expressed based on (i) molecular adsorption competition resulting in phase separation and (ii) after thermal mediation, host–guest attractions leading to thermodynamically preferred co-crystals. Also, in the literature, host–guest geometric matching may lead to structural locking.^36,37^ However, we showed that a mismatching case might also yield co-crystals provided that suitable conditions were met. The temperature effect on the host can be expressed in terms of molecular polymorphs and their lattice structures. On the other hand, the guest could also be influenced, *i.e.* conformations, which however did not change the resulting outcomes. The important finding unveiled here is that temperature (thermal annealing effect) not only affects individual molecular self-assembly but also influences the hetero–molecular attractions, determining phase-separated or co-crystal outcomes of a binary molecular system at surfaces.

**Fig. 5:**
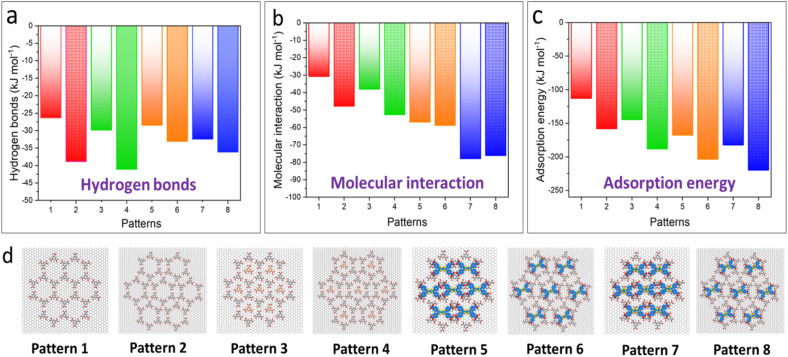
Force field calculation results of (a) hydrogen bonds, (b) molecular interaction and (c) adsorption energies. For every histogram, data in the same color without (patterns 1, 3, 5, and 7)/with (patterns 2, 4, 6, and 8) shape-filling represent the TMA-CW and TMA-flower framework, respectively. (d) Models of patterns 1–8.

Finally, the energy landscape unveiled the stability of these polymorphic structures of TMA and the TMA–TTF co-crystals (see Fig. S9 and S10 SI). Higher temperatures for thermal annealing led to more stable packing (more negative adsorption energy). The packing models are presented in Table S1 in the SI. The lower value of its total energy per unit area represents higher stability. Hydrogen bonding, molecular interactions and adsorption energy were calculated, as shown in Fig. 5. It is clear that regardless of whether the underlying template is self-trapped or not, the flower type co-crystals (patterns 2, 4, 6, and 8) are more stable than the CW networks (patterns 1, 3, 5, and 7). Note that in Fig. 5, for molecular interactions, the CW and flower frameworks of patterns 7 and 8 show minimal differences (−77.8 and −76.0 kJ mol^−1^, respectively). Considering the overall self-assembly process (hydrogen bonds and adsorption energy), the flower type is believed to be more stable than the CW type. During our STM observations, the flower type co-crystal network is indeed the most stable one, which consistent well with the calculation results.

By employing temperature as a tool for kinetic enhancement and thermodynamic guidance, researchers are able to better tailor molecular self-assembly processes. In general, elevating temperature can influence the molecular self-assembly process by modulating the energy landscape of intermolecular interactions, leading to a reduction in energy barriers associated with molecular diffusion, conformational changes, and surface adsorption. Therefore, molecular self-assembly is fundamentally a thermodynamically governed process. However, its kinetics—how rapidly and effectively molecules reach their equilibrium configurations—are critically dependent on external conditions such as temperature. At elevated temperatures, the increased thermal energy provides molecules with greater mobility, enabling them to overcome kinetic traps and local energy minima that would otherwise hinder the formation of well-ordered supramolecular structures. Also, this thermal activation can facilitate more efficient molecular rearrangements, allowing the system to explore a wider configuration space and potentially access the global minimum of the free energy landscape. In systems where self-assembly is limited by diffusion-limited aggregation or by the slow conformational adaptation of flexible molecular backbones, heating can significantly accelerate the assembly process and result in higher structural uniformity and long-range order.

## Conclusions and outlook

In summary, we have demonstrated that thermal stimuli can offer a straightforward means to modulate binary supramolecular co-assembly. The application of heat enables the formation of co-crystals that would otherwise result in phase separation under ambient conditions, as studied by STM at the liquid–solid interface. This is attributed to the inherent capacity of the molecular components to competitively adsorb and self-assemble on the HOPG surface, where host–guest interactions and cooperative assembly dynamics can be finely tuned through thermodynamic regulation. The externally applied heat acts as a decisive factor in guiding these processes, serving, for example, as a mediator in this work. We envision that by leveraging temperature as both a kinetic accelerator and a thermodynamic modulator, this method may offer significant potential for tailoring co-assembly pathways in a wide range of scientific and technological applications. For the outlook, the current host–guest vertical stacking may outperform conventional co-assemblies, such as the TMA–coronene system where both the host and guest molecules are in the same plane. In the present system, the self-trapped TMA can further be replaced, for example, by TCNQ, potentially leading to the development of molecularly precise donor–acceptor nanoscale rectifiers or surface-confined reactors.^38,39^

## Author contributions

F. Chen, J. He, and A. Shaheen performed STM studies and analysed the data, and contributed to writing the manuscript. Y. Hu analysed the data, performed the force field calculations, and contributed to writing the manuscript. S.-L. Lee conceived the idea, supervised the project, and contributed to writing the manuscript.

## Conflicts of interest

There are no conflicts to declare.

## Supplementary Material

SC-017-D5SC06698K-s001

## Data Availability

The data that support the findings of this study are available from the corresponding authors upon reasonable request. Supplementary information (SI) is available. See DOI: https://doi.org/10.1039/d5sc06698k.
