# Supplementary material for: Thermal-mediated modulation of binary supramolecular self-assembly from phase separation to co-crystallization at the liquid–solid surface
Source: Chem Sci. 2026 Jan 13;17(9):4715–21. doi: 10.1039/d5sc06698k (PMC12797247; doi:10.1039/d5sc06698k)
Supplement: SC-017-D5SC06698K-s001 [file SC-017-D5SC06698K-s001.pdf]

Supplementary Information (SI) for Chemical Science.
This journal is © The Royal Society of Chemistry 2026

Thermal-mediated modulation of binary supramolecular self-assembly from phase separation to co-crystallization at the liquid–solid surface

Fang Chen,[a] Jun He,[a] Attia Shaheen,[a] Yi Hu,[b,*] and Shern-Long Lee[a,*]

a. Institute for Advanced Study Shenzhen University, Shenzhen, Guangdong, 518060, China.
b. School of Chemistry and Chemical Engineering, Anqing Normal University, Anqing, Anhui 261433, China.

*E-mail: sllee@szu.edu.cn; hu.yi@aqnu.edu.cn

Figure S1: STM monitoring of the dynamic process of layered TTF.
Figure S2: A histogram of the onset times of TMA and TTF self-assembly formations.
Figure S3: A surface obtained by the layer-by-layer deposition of TMA and TTF.
Figure S4: Apparent high difference of the TMA-TTF co-crystal.
Figure S5: Concentration effect for surface TMA-TTF host-guest polymorphs.
Figure S6: Solvent effect for surface TMA-TTF host-guest polymorphs.
Figure S7: The polymorphs for TMA polymorphs on the graphite surface.
Figure S8: Thermal effect for TMA polymorphs on the graphite surface.
Figure S9: An STM image to show the TMA template of TMA-TTF co-crystals.
Figure S10: The dynamic equilibrium of the TMA-TTF co-crystal at the liquid-solid interface.

STM experiments:

Force field calculations:
Table S1. Calculation details of different patterns.

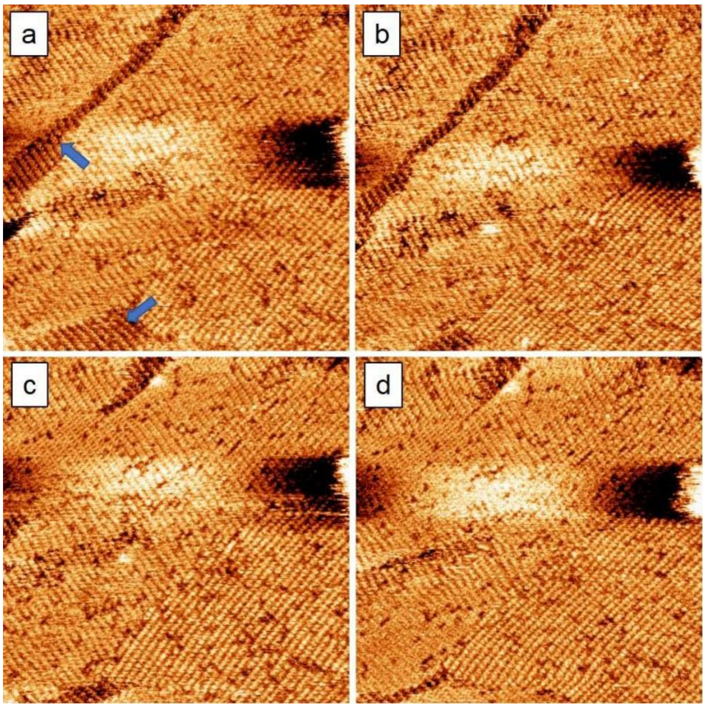

Figure S1: STM monitoring of the dynamic process of layered TTF. The time-dependent STM images of the TTF surface was prepared by a high concentration sample (0.1 M). STM monitoring revealed the dynamics where monolayers (as indicated by arrows) were gradually replaced by bilayers. The dynamics suggested that the monolayers were unstable and the meta-stable phases gradually disappeared during the scanning. The images were consecutively captured and each panel (70×70 nm^2) took ca. 4 mins. Imaging conditions: E_{bias}, -0.85 V; $i_{\text{tunneling}}$, 350 pA.

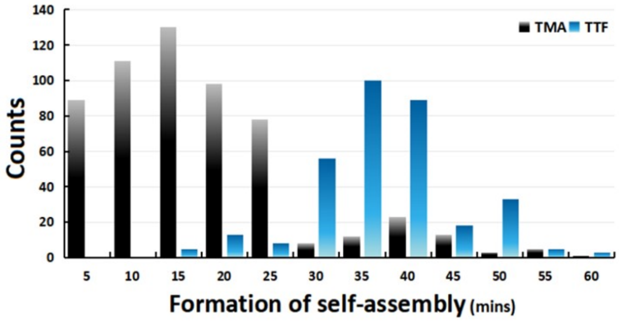

Figure S2: A histogram of the onset times of TMA and TTF self-assembly formations. The histogram was obtained by STM imaging following individual sample solution depositions (0.1 M with OA as the solvent); they were recorded across 15 independent experimental sessions. The results suggest that the formation rate of TTF self-assembly is lower than that of TMA. Note that to increase solubility of TMA and TTF yet avoid thermal effect on the system, a sonication at a gentle temperature (25℃, 30 mins) was used. The TMAs presented higher kinetics compared with that of TTF because their self-assemblies appeared faster. For the co-assembling, the surface results were determined via firstly kinetic control followed by thermodynamic one. The TTF became the majority of the surface and its occurrence depended on the TTF concentrations. The higher concentrations of TTF were used, the faster the surface was occupied by TTF. Moreover, the co-crystal yielded only after heating treatments, emphasizing the importance of thermal stimuli.

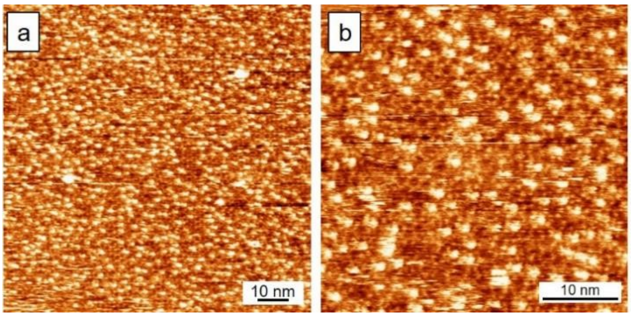

Figure S3: A surface obtained by the layer-by-layer deposition of TMA and TTF (OA as the solvent). Imaging conditions: E_{bias}, -0.85 V; $i_{tunneling}$, 350 pA. The sample solutions were without thermal treatments. These TTFs tended to pack into dimers or randomly adsorbed atop the honeycomb template of TMA, emphasizing the importance of thermal stimuli in the system.

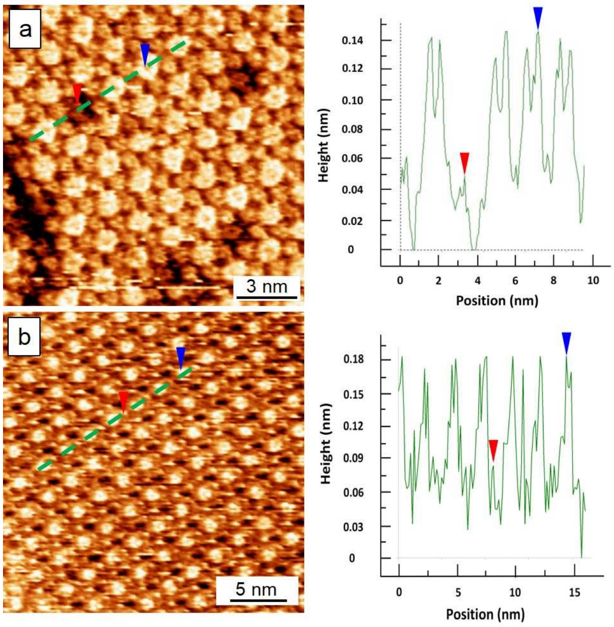

Figure S4: Apparent high difference of the TMA-TTF co-crystal. (a, b) refer to the CW and flower types co-crystals. The high differences of both types are ca. 0.1 nm, indicating bilayers. The red and blue arrows indicate the two layers on images and their corresponding cross-sectional profiles. Imaging conditions for a): E_{bias}, -0.8 V; $i_{tunneling}$, 550 pA and b): E_{bias}, -0.85 V; $i_{tunneling}$, 300 pA.

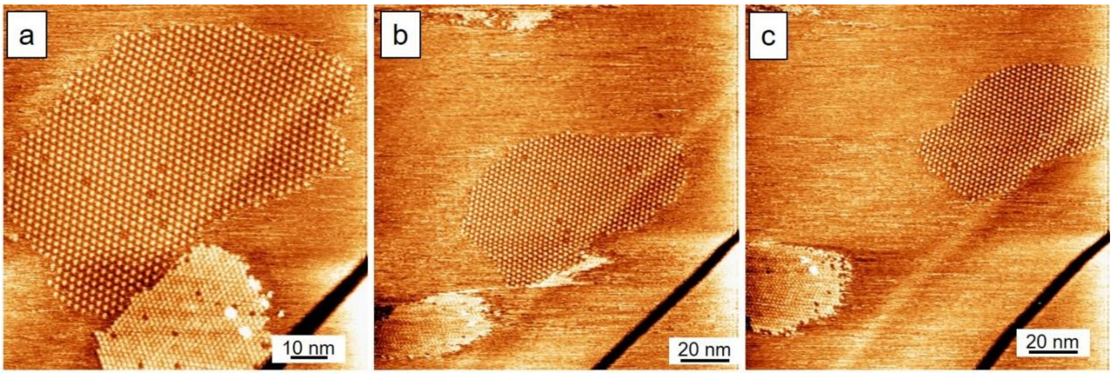

Figure S5: Concentration effect for surface TMA-TTF host-guest polymorphs. For lower concentrations, neither TMA nor TTF was able to self-assembly stably on the surface. However, upon heating, the surface appeared sub-monolayer co-crystals. This implies that the occurrence of co-crystals was less related to sample concentration but depended largely on its temperature. The sample surface was formed by low concentration (0.01 M, OA) after thermal treatments (80°C, 30 min). These images also revealed that the chicken-wire cocrystal (the lower island) is relatively unstable compared with the flower type, consistent with our force field simulations. The images were captured 15 mins for each panel. Imaging conditions: E_{bias}, -0.85 V; $i_{tunneling}$, 300 pA.

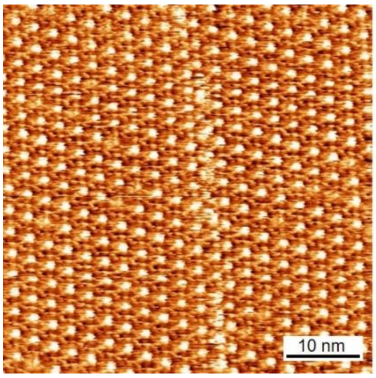

Figure S6: Solvent effect for surface TMA-TTF host-guest polymorphs. Imaging conditions: E_{bias}, -0.85 V; $i_{tunneling}$, 300 pA. The sample was prepared by HA (0.1 M) and heated to 50°C for 30 min. After gentle thermal annealing of the surface, the flower-type co-crystal is the dominant packing, emphasizing solvent effect (c.f. with Fig 3 in the maintext, CW-type co-crystal is exclusively formed using OA as the solvent). The STM image was taken at room temperature.

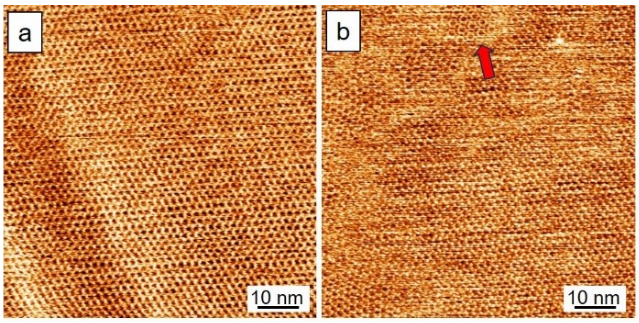

Figure S7: The polymorphs for TMA polymorphs on the graphite surface. Occasionally, a small domain of flower pattern appeared during STM scanning at RT (the red arrow). However, it disappeared after the Ostwald ripening process. Imaging conditions: E_{bias}, -0.55 V; $i_{tunneling}$, 300 pA.

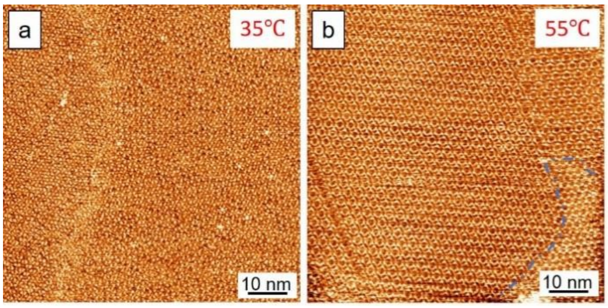

Figure S8: Thermal effect for TMA polymorphs on the graphite surface. Imaging conditions: E_{bias}, -0.55 V; $i_{tunneling}$, 300 pA. The results show that thermal effect can influence the polymorphic packing of TMA. The partial self-trapped chicken-wire (CW) network was observed at 35℃. The more stable flower packing appeared at higher temperatures and its domain size varied as a function of temperature. The flower polymorph was exclusively formed at 60℃, which is lower than that of the flower type TMA-TTF co-crystal. This indicates that higher energy is needed for overcoming the energy barrier toward co-crystal yielding. The patterns were obtained at room temperature suggesting that once formed, they were stable on the surface.

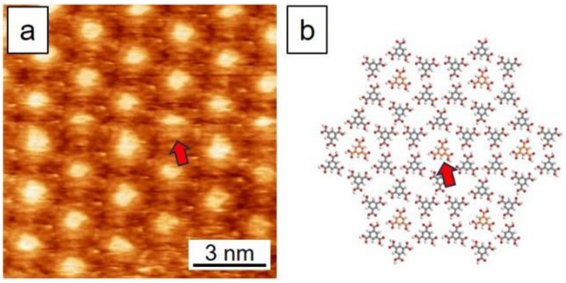

Figure S9: An STM image to show the TMA template of TMA-TTF co-crystals. Imaging conditions: E_{bias}, -0.85 V; $i_{tunneling}$, 300 pA. The self-trapped TMA as indicated may contribute extra energy to stabilize the co-crystal. The gained energy was estimated by force field simulations.

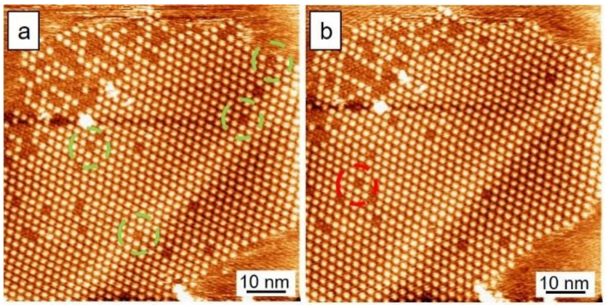

Figure S10: The dynamic equilibrium of the TMA-TTF co-crystal at the liquid-solid interface. The green and red defects disappeared and appeared respectively, indicating that the equilibrium at the liquid-solid interface was dynamic and the surface underwent self-repairing (less influenced by STM scanning). The sample surface was formed by low concentration (0.01 M, OA) after thermal treatments (80℃, 30 min). Imaging conditions: E_{bias}, -0.85 V; $i_{tunneling}$, 300 pA.

STM experiments

The STM experiments were performed at the OA(or HA)/HOPG interface using a Bruker-MS10 operating in the sample-biased and constant-current mode at room temperature (*ca.* 23 °C). The experiments were repeated several times. STM tips were mechanically cut Pt/Ir wires (80%/20%, diameter 0.25 mm). HOPG was purchased from Advanced Ceramics (ZYB grade, Advanced Ceramics Inc.). Imaging conditions of V_{bias} and I_{set} were ranged from −1.50 to 1.50 V and from 300 to 500 pA, respectively. The reported STM images were calibrated by the unit-cell vectors of the underlying HOPG using an SPIP software (scanning probe image processor, Image Metrology ApS). The chemicals were commercially available (Jilin Chinese Academy of Sciences-Yanshen Technology Co., Ltd.) and were used as received, including tetrathiafulvalene (TTF, ≥95%); trimesic acid (TMA, ≥98%), and octanoic/heptanoic acid (OA/HA, ≥ 99%). The modelled molecular packing structures were obtained using HyperChem™ Professional 7.5 program based on the lattice-structure parameters. First, a molecular model for a single molecule was built, and then this model was duplicated. We constructed the model of the entire monolayer *via* placing the molecules in accordance with the intermolecular distances and angles obtained from calibrated STM images. The imaging parameters are indicated in the figure caption: sample bias (E_{bias}) and tunnelling current (I_{set}). In these experiments, the temperature was controlled by heating plate, sonication machine, and directly drop-casting a warm solvent onto the surface. The co-crystals were stable after thermal treatments. They were imaged at room temperature (RT).

Force field calculations:

To gain further insight, we performed force field simulations on the hydrogen bond (ΔE_H), van der Waals interaction (ΔE_V), and adsorption energy (ΔE_{ads}) of these molecular networks (Table S1). All simulations and calculations were performed using the Materials Studio software, using the Dreiding force field. This force field was selected because it is able to properly describe the intermolecular interactions and the hydrogen bond. The adsorption energy was calculated with a piece of graphene to represent the substrate.

Calculation details:

Setup:

Task: Geometry Optimization

Quality: Ultrahigh

Energy:

Forcefield: Dreiding

Charges: Charge using Gasteiger

Quality: Ultrahigh

Electrostatic: Atom based

Convergence Tolerance:

Quality: Ultrahigh

Energy: $2.0e^{-5}$ kcal/mol

Force: 0.001 kcal/mol/Å

Displacement: $1.0e^{-5}$ Å

Max interactions: 5000

Note: HOPG substrate was constrained in place during the optimization.

$\mathbf{E_{system}}$: the total energy of the system.

$\mathbf{E_{molecule}}$: the energy of the molecule.

$\mathbf{N_{sum}}$: total number of molecules used for the calculation

$\mathbf{E_{substrate}}$: the energy of the substrate.

S: area of the model used for the calculation

For the binding energy of hydrogen bond and molecular interaction:

$$\mathbf{\Delta E = (E_{system} - N_{sum}E_{molecule})/S}$$

For adsorption energy:

$$\mathbf{\Delta E = (E_{system} - N_{sum}E_{molecule} - E_{substrate})/S}$$

The energy details of different patterns are shown as follows, in Table S1.

Table S1. Calculation details of different patterns.

Patter Number	Pattern model	Number of TMA	Number of TTF	Total area S(nm^2)	Hydrogen bond (kJ mol^{-1})	Molecular interaction (kJ mol^{-1})	Adsorption energy (kJ mol^{-1})
Pattern-1	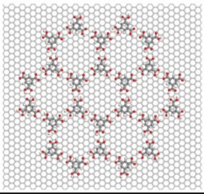	24	0	30.33	−26.3	−30.7	−112.7
Pattern-2	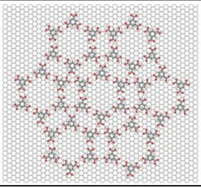	42	0	37.89	−38.8	−47.7	−158.0
Pattern-3	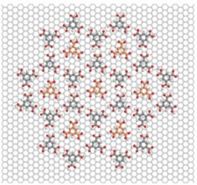	31	0	30.33	−29.8	−38.0	−144.5
Pattern-4	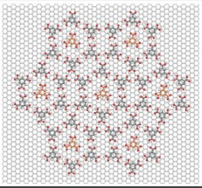	49	0	37.89	−41.0	−52.6	−188.1
Pattern-5	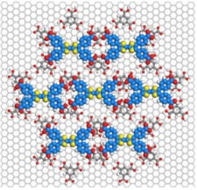	24	7	30.33	−28.4	−56.8	−167.7
Pattern-6	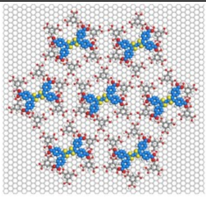	42	7	37.89	−33.0	−58.7	−203.3

Pattern-7	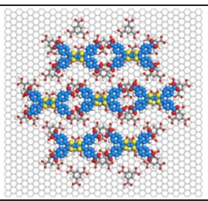	31	7	30.33	−32.4	−77.8	−182.2
Pattern-8	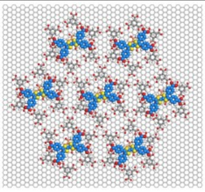	49	7	37.89	−36.1	−76.0	−219.8
Pattern-9	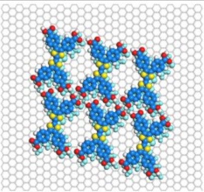	0	12	15.84	−19.7	−72.2	−182.4
